# Endometriosis Is a Cause of Infertility. Does Reactive Oxygen Damage to Gametes and Embryos Play a Key Role in the Pathogenesis of Infertility Caused by Endometriosis?

**DOI:** 10.3389/fendo.2018.00725

**Published:** 2018-11-29

**Authors:** Gábor Máté, Lori R. Bernstein, Attila L. Török

**Affiliations:** ^1^Pannon Reproduction Institute, Tapolca, Hungary; ^2^Pregmama, LLC, Gaithersburg, MD, United States; ^3^Department of Epidemiology and Public Health, School of Medicine, University of Maryland, Baltimore, MD, United States; ^4^Department of Veterinary Integrative Biosciences, Texas A&M College of Veterinary Medicine, College Station, TX, United States

**Keywords:** oocyte, aneuploidy, embryo, endometriosis, infertility, reactive oxygen species, sperm

## Abstract

Approximately, 10–15% of women of reproductive age are affected by endometriosis, which often leads to infertility. Endometriosis often has an inherited component, and several causative predisposing factors are hypothesized to underlie the pathogenesis of endometriosis. One working hypothesis is the theory of retrograde menstruation. According to the theory of retrograde menstruation, components of refluxed blood, including apoptotic endometrial tissue, desquamated menstrual cells, lysed erythrocytes, and released iron, induce inflammation in the peritoneal cavity. This in turn activates macrophage release of reactive oxygen species (ROS), leading to oxidative stress via the respiratory burst. Refluxed blood promotes the Fenton reaction, terminating in the production of hydroxyl radical, the most potently destructive ROS. In this article, we review the papers that demonstrate decreased quantity and quality of oocytes and embryos retrieved from IVF/ICSI patients with endometriosis. We discuss literature data demonstrating that ROS are generated in endometriotic tissues that have physical proximity to gametes and embryos, and demonstrating adverse impacts on oocyte, sperm and embryo microtubule apparatus, chromosomes, and DNA. Data that addresses the notions that endometriosis causes oocyte and fetal aneuploidy and that these events are mediated by ROS species are also discussed. Literature data are also discussed that employ use of anti-oxidant molecules to evaluate the importance of ROS-mediated oxidative damage in the pathogenesis of endometriosis. Studies are discussed that have employed anti-oxidants compounds as therapeutics to improve oocyte and embryo quality in infertile subjects, and improve fertility in patients with endometriosis.

## Introduction

Endometriosis is a disease in which endometrial cells migrate outside the uterine cavity and form “implants” that colonize in distal tissues. These include but are not limited to the fallopian tube, the ovary, and peritoneum (1). Endometrial implants are estrogen-dependent for their growth (2). Endometriosis is a frequent finding among infertility patients. Often it takes years before a patient knows she has it. Accurate statistics are not available as to the actual the frequency of the disease. It is commonly accepted that 10 to 15% of reproductive age women are suffering from endometriosis. Twenty-Five to Fifty percent of female patients undergoing fertility treatments are being treated for endometriosis (3).

In infertile patients, an exact staging of endometriosis is very important. The American Society of Reproductive Medicine (ASRM) classification system is used world-wide. The classification is based on laparoscopic findings. Number, location and size of the endometriotic implants are drawn on a figure of the pelvis and degree of adhesions and lesions and the place of the endometriotic tissues are scored. The classification of endometriosis is based on this point score (4). Generally, ASRM I endometriosis is defined by only superficial lesions and possibly a few adhesions, ASRM II endometriosis is defined by some deep lesions in the recto-uterine pouch, ASRM III is defined by the presence of endometriotic tissues on the ovary resulting endometriotic cysts called endometriomas. ASRM IV is defined by large endometriomas with extensive adhesions or deep infiltrating the bowels, the bladder and/or retroperitoneal tissues. Surgical treatments are performed to directly remove endometriotic implants from extrauterine locations. Medical treatments are comprised of long-term GnRH suppression with GnRH agonists to shrink existing endometriotic implants and suppress the growth of new ones. IVF is also used to abrogate infertility caused by endometriosis. For patients with ASRM I and II endometriosis there is a good prognosis for successful infertility treatment using either surgical, or medical treatment and/or assisted reproductive techniques (ART). While there is a 60–70% cumulative clinical pregnancy rate for ASRM I/II patients undergoing ART, the other treatments could also be successful and to date there is no unified view as to what kind of therapy should be recommended for these patients (5). On the other hand, there is now unanimous consensus for stages III and IV that only ART is effective (6). Even so, ART success rates in Stage III/IV patients remain significantly lower than for stage I/II patients (5).

Here we provide an overview of the pathogenesis of endometriosis. We then discuss current and emerging understandings of the pathologic mechanisms by which endometriosis causes infertility. We present that endometriosis induces decreased quantities of oocytes and embryos, decreased embryo quality, and decreased implantation and pregnancy rates in patients with endometriosis. We then discuss evidence that highly reactive free radical species, especially reactive oxygen species (ROS) generated in endometriosis patients damage intracellular structures and genomic material to adversely impact the structural integrity and viability of oocytes, sperm, and embryos, evaluate literature data that implicates oocyte and fetal aneuploidy as contributing factors for infertility caused by endometriosis, and we discuss therapeutic horizons for treatment of endometriosis patients with antioxidant therapies.

### Part I: overview of the pathogenesis of endometriosis

Most frequently, endometriotic implants are found in the pelvis. The common sites of endometriotic implants include the surfaces of the ovaries, fallopian tubes, and pelvic peritoneum, all of which have physical proximity to ovarian follicles. The uterosacral ligaments are also common implant sites (alternatively called the “pelvic” sites) (7). Atypical sites for endometriosis include the gastrointestinal tract, urinary tract, soft tissues, and within the thoracic cavity including the pleura and the lungs (8, 9).

#### Retrograde menstruation

The most frequent accepted theory that explains the pathogenic process by which these lesions occur is the theory of retrograde menstruation, the theory that the backup of extrinsic menstrual tissues into the fallopian tubes, pelvis, and peritoneum is a driving force in the formation of endometriomal implants. From the study of Halme et al. (10) is well known that 90% of women with patent tubes have peritoneal fluid (PF)/blood in the cul-de-sac during the menstrual period, irrespective of whether they are healthy or suffering from endometriosis. PF is present only in 15% of patients with tubal occlusion. From these data it is evident that the occurrence of retrograde menstruation alone is not sufficient to explain the development of endometriosis. Instead a multifactorial mechanism is likely. This theory helps explain the occurrence of abdominal-pelvic-peritoneal endometriosis, but it does not explain the atypical forms of endometriosis.

#### Coelomic metaplasia and metastatic spread

In 1969, Ferguson et al. demonstrated Müllerian metaplasia of coelomic epithelia (11). The presence of metaplastic epithelia was also found in the pelvic lymph nodes. This is an evidence that beside the peritoneum other organs may also contain undifferentiated cells. Based on this finding endometrial cell transformation from these cells is theoretically possible. With this theory, not only the pelvic peritoneal forms of endometriomas, but also the atypical endometriomas can be explained.

#### Altered immunity

When endometrial tissue relocates to the abdominal cavity—as in the case of retrograde menstruation—both the cellular and humoral immune systems are activated (12). In patients with endometriosis the cellular mediated immune reaction does not recognize the endometrial tissue as “foreign.” This is permissive for endometrial cells to implant in the peritoneum (13, 14). After implantation, macrophage and leukocyte invasion are detected in the surroundings of the implants and in the PF. These cells secrete cytokines and growth factors, including IL1, IL-6, IL8, TNF, VEGF, CCL5, RANTES; and other cytokines (14–16). This complex network of locally produced cytokines stimulates the growth and inflammatory behavior of the ectopic endometrial implants. Proinflammatory cytokines secreted from endometriotic lesions enhance the inflammatory reactions that occur in endometriosis. They promote survival and growth of these lesions and block their demise (17).

#### Stem cells

After menstruation, the endometrium regenerates from the lower basal layer. The basal layer contains adult stem cells and a small population of epithelial and stromal stem cells (18). Recently, Cousins and Gargett (19) identified several types of stem/progenitor cells in the basal layer of the endometrium. It is logical to postulate that these types of cells could also reach the abdominal cavity during the menstrual period via retrograde menstruation, to thereby play a key role in the pathogenesis of endometriosis. The notion has also been advanced that non-endometrial stem cells, such as blood-borne bone marrow derived cells, may also reach the abdominal cavity, differentiate into endometrial cells, and implant in the peritoneum and the surfaces of organs in the abdominal cavity (20).

#### Genetics and altered gene regulation in endometriosis

Familial inheritance of endometriosis has been widely reported in the literature (21). Saha et al. (22) reported that the incidence of endometriosis in monozygotic twins is double that of dizygotic ones. Based on these data, the best-fitting model revealed a contribution of 47% by additive genetic factors and the remaining 53% attributed to environmental effects. More recently, based on meta-analysis of 17,045 endometriosis cases, Fung and Montgomery (23) suggested that the most common genetic factors contributing to endometriosis risk are located in regulatory DNA sequences that control gene transcription. Genes with altered gene regulatory sequences include LINC00339, VEZT CDC42, and CDKN2A-AS1. CDC and CDK genes encode genes that regulate cell cycle progression. It is logical to hypothesize that dysregulation of their expression may promote endometrial growth. This observation is most consistent with a polygenic/multifactorial etiology, or via causation by several alternate pathways, although alternative mechanisms cannot be excluded.

### Part II: how does endometriosis cause infertility?

#### Patients with endometriosis have decreased quality and quantity of oocytes and embryos, decreased implantation and pregnancy rates, and increased rates of spontaneous abortion

Patients suffered from endometriosis showed lower ovarian reserved (24). As an indicator of this phenomenon, significantly altered level of serum AMH was reported by Seyhan et al. (25) in endometriosis patients than healthy controls. In contradiction with this, Kucera et al. (26) observed no differences in follicular fluid (FF) AMH levels between women with endometriosis and healthy control women. Several investigators have observed decreases in fertilization rate, yields of high-quality day 3 embryos, rates of blastocyst formation, and rates of implantation and pregnancy in IVF endometriosis patients compared to control subjects, and also in a mouse model of endometriosis embryotoxicity (24, 27–30). A concomitant increase in the rate of spontaneous abortions is also observed (24). Moreover, the severity of alterations depends on the stage of endometriosis: patients with Stage III-IV endometriosis have fewer retrievable oocytes, fewer fertilizable and fertilized oocytes, fewer embryos poorer quality embryos, lower rates of implantation, and fewer fetuses than the group of patients with Stage I-II endometriosis or without endometriosis. The published decrement in the embryological parameters is probably the consequence of oxidative stress processes leading to genetic instability. This will be explained in details in the further sections.

#### Multiple factors in the pathogenesis of endometriosis-associated infertility: an overview

##### Mechanical factors

Multiple factors have been implicated as contributing to infertility of patients with endometriosis. Mechanical factors play important roles. Occlusion of the fallopian tubes and peritoneal adhesions impede fertilization and implantation by mechanically blocking the transfer of oocytes, sperm, and embryos through the fallopian tubes. In addition, gamete transfer is inhibited due to impedance of tubal motility due to elevated levels of cytokines (31).

##### Hormones

Hormonal status is altered significantly in patients with endometriosis. Endometriosis is associated with an increased aromatase enzyme activity in granulosa cells, resulting in elevated follicular estradiol secretion (32). While normal menstrual cycle levels of estradiol promote the healthy development and receptivity of the endometrium, the high estradiol levels of endometriosis patients elicit pathologic changes in the eutopic proliferative and secretory endometrium. Elevated estradiol is also key to the pathogenic process of endometriosis because it drives the growth of extrauterine endometriotic implants.

Endometriosis patients frequently have elevated prostaglandins (33). This causes uterine contractions, which are detrimental to the maintenance of pregnancy. Hyperprolactinemia is another frequent finding, although the cause is not yet clear. Hyperprolactinemia causes corpus luteum dysfunction, which increases rates of spontaneous abortion, the rates of which are significantly higher in patients with endometriosis (34).

##### HOXA10

The homeobox genes encoding HOXA10/HOXA 10 transcription factors have key roles in embryogenesis, endometrial regeneration, and endometrial receptivity (35). Patients with endometriosis exhibit a failure to increase expression of HOXA10 in the mid luteal phase, at the peak of the implantation window. This is correlated with increased rates of implantation failure.

##### Endometrial receptivity vs. egg quality

Elevations in estradiol, prostaglandins, HOXA10 expression, and prolactin, as well as progesterone resistance (36) may reduce endometrial receptivity in endometriosis patients, thereby impeding implantation and contributing to their infertility (32, 34, 35). However, while implantation rates are clearly diminished, it is still unknown which impairments in endometrial receptivity are causative factors for infertility in endometriosis patients. In a key study, Simón et al. reported that donor egg recipients with endometriosis receiving eggs from fertile donors had the same rates of implantation and pregnancy as recipients without endometriosis (37). Conversely, donor egg recipients who received eggs donated by patients with endometriosis had significantly reduced implantation rates compared to other patient groups. These data indicate that compromised egg and embryo quality rather than endometrial receptivity plays a principal role in diminished rates of implantation and fecundity in patients with endometriosis.

##### Immune and inflammatory factors

Immune and inflammatory factors are thought to play key roles in the pathogenesis of infertility in patients with endometriosis, by reducing gamete quality and rates of gamete transport and implantation, and by increasing rates of pregnancy loss. Invasion of macrophages and other leukocytes into the PF and the vicinity of endometriotic implants are thought to play an important role in the pathogenesis of endometriosis infertility by releasing potent, highly reactive free radical species that directly damage sperm, oocytes, and embryos (14). Elevated levels of inflammatory cytokines, growth- and angiogenic factors have toxic effects on sperm, oocytes, embryonal development, gamete transportation, and implantation. Elevated levels of anti-endometrial antibodies are detectable in the blood serum and PF from patients with endometriosis compared to healthy controls. This is correlated with increased frequencies of miscarriages (38). It is reasonable to hypothesize that that these antibodies attack the integrity of the endometrium by compromising sustained implantation and increasing the likelihood of spontaneous abortion. In addition, immune cells generate ROS during this inflammatory process through the respiratory burst NADPH oxidase system (39). Levels of ROS produced by these inflammatory responses in the environments of endometriomal implants are significantly higher in comparison with their healthy counterparts (40). The pathological increase in NADPH oxidase-mediated superoxide (O${}_{2}^{•-}$) and peroxide production from inflammatory phagocytic cells in the course of this inflammatory reaction acts as source of oxidative stress. Not only the oxidative burst is responsible for oxidative stress. From the refluxed blood, erythrocytes may lose their integrity and iron releases. In the presence of this kind of transition metals other ROS can be produced (29).

### Part III: oxidative stress and its role in infertility in patients with endometriosis

#### Overview of oxidative stress and the antioxidant response

Free radicals are ions, atoms or molecules containing a free unpaired electron in the outer electron shell. The free radical state is highly unstable, thus highly reactive in capturing electrons from other molecules. Levels of free radical species in healthy tissues exert important intracellular signal transduction and regulatory functions in folliculogenesis, maturation of oocytes, dissolution of corpora luteal, implantation, and embryo development (40, 41). While free radical species are normal by-products of cellular metabolism, they are produced copiously in the context of inflammation. Due to their chemical instability and reactivity, free radicals in excess do indiscriminate damage to cellular organelles and molecular components, including DNA, RNA, proteins, lipids, carbohydrates, and their building blocks (nucleotides, amino acids, etc.). This results in aberrations in cellular structure and function, and mitotic and meiotic inheritance, and even cell death. Therefore levels of free radical species are closely controlled by endogenous antioxidant molecules that quench their reactivity. The most biologically important types of free radicals in mammalian systems are “reactive oxygen species” (ROS). “Oxidative stress” occurs in cells when there is an unbalanced state of ROS excess compared to available antioxidant activity. This can be caused by excessive production or exposure to ROS, inhibition of antioxidant synthesis, or depletion of antioxidants (42).

#### Reactive oxygen species *in vivo*

The predominant ROS species are O${}_{2}^{•-}$, hydrogen peroxide (H_2_O_2_), and, hydroxyl radical (^•^OH) (43). These potent ROS are predominantly the products of incomplete reduction of molecular oxygen in the mitochondrial electron transport system, where electrons leak from the NADH-ubiquinone oxidoreductase to molecular oxygen (44).

##### Superoxide anion

Approximately, 1–5% of molecular oxygen is converted into O${}_{2}^{•-}$ by mitochondrial complexes during normal respiration (45). O${}_{2}^{•-}$ is a highly reactive radical that does significant intracellular damage. O${}_{2}^{•-}$ is capable of oxidizing cytochrome c in the respiratory chain. It can oxidize polyphenols, tocopherol, and thiol (i.e., cysteine, methionine). Furthermore O${}_{2}^{•-}$ may be able to inactivate catalase (CAT) a major antioxidant enzyme that is most abundant in peroxisomes.

O${}_{2}^{•-}$ is able to either reduce or oxidize transition metals (e.g., iron, copper) (46), which function as catalysts for redox reactions that produce reactive oxygen species. Through the reduction of Fe(III) shown in Reaction 1, O${}_{2}^{•-}$ destroys the Fe-S clusters of proteins, and the reduced iron undergoes additional redox reactions that cause more injuries, as described below (44).

(1) $$\begin{array}{r} \text{F}{\text{e}}^{3+}+{{\text{O}}_{2}}^{•-}⇄\text{F}{\text{e}}^{2+}+{\text{O}}_{2} \end{array}$$

(2) $$\begin{array}{r} \text{F}{\text{e}}^{2+}+2{\text{H}}^{+}+{{\text{O}}_{2}}^{•-}⇄\text{F}{\text{e}}^{3+}+{\text{H}}_{2}O_{2} \end{array}$$

##### Hydrogen peroxide

As shown in Reaction 2, Fe(II) returns to the oxidized Fe(III) state, in the process catalyzing the production of H_2_O_2_. Aerobic respiration is also a major source of H_2_O_2_ in mitochondria. Every system that generates O${}_{2}^{•-}$ also produces H_2_O_2_ via a dismutation reaction by the enzyme superoxide dismutase (SOD). Mitochondrial respiration and peroxisomal lipid metabolism are the primary sources of H_2_O_2_ in eukaryotic cells (47). H_2_O_2_ is highly reactive in its own right. It forms adducts with various cellular components. It reacts with cysteine and methionine amino acid side chains causing protein damage. However, the greatest damage that is done by H_2_O_2_ derives from its ability to form hydroxyl radical ^•^OH, the most pernicious and reactive of the ROS species.

##### Hydroxyl radical

The reactivity of O${}_{2}^{•-}$ and H_2_O_2_ are significantly lower than that of ^•^OH. Intracellular production of ^•^OH from H_2_O_2_ is driven by the Fenton reaction, shown in Reaction 3 (43, 44, 48):

(3) $$\begin{array}{r} \text{F}{\text{e}}^{2+}+{\text{H}}_{2}O_{2}⇄\text{F}{\text{e}}^{3+}{+}^{•}\text{OH}+\text{O}{\text{H}}^{-} \end{array}$$

As the most reactive known ROS, ^•^OH reacts with nearly all intracellular building blocks and macromolecules (amino acids, nucleic acids, phospholipids, sugars, proteins, RNA, DNA, lipids, polysaccharides). Hence the physiological concentration in cells is about zero. No enzymatic defense against ^•^OH is known. The only protection is realized via the strict control of iron metabolism (49).

### Antioxidant defenses

Antioxidants comprise the principal means of intracellular protection from free radical damage (48). The term “antioxidant” is used to describe molecules that directly react with and inactivate ROS species. The term also refers to molecules that function indirectly, either by activating downstream antioxidant protection systems, or by inhibiting pro-oxidant systems that generate ROS. The antioxidants are in two major groups: non-enzymatic systems, and enzymatic systems.

#### Non-enzymatic-defense

The glutathione tripeptide molecule (GSH; L-Υ-glutamyl-L-cysteinylglycine) is a key peptide component of intracellular anti-oxidant defense (50). Other classes of non-enzymatic components include vitamins (e.g., ascorbic acid, α-tocopherol), enzymatic cofactors (e.g., Coenzyme Q10), nitrogen compounds (e.g., uric acid), carotenoids (e.g., ß-carotene, lutein), and minerals (e.g., zinc, selenium). There are two forms in cells: reduced (GSH) and oxidized (glutathione disulfide: GSSG). GSH protects cells against oxidative stress by hydrogen or electron donation. The ratio of these two forms is a good indicator of oxidative stress. GSH serves as a cofactor for several detoxification enzyme (e.g., glutathione peroxidase: GPx), assists in the transport of amino acids through the plasma membrane, and is able to regenerate the C- and E-vitamins and direct scavenger of ^•^OH and singlet oxygen (42, 47). GSH is a “universal” antioxidant molecule, because it can quench nearly every kind of ROS, including O${}_{2}^{•-}$, H_2_O_2_, ^•^OH, and peroxidized lipids (51, 52). The balance between GSH and GSSG is maintained by GSH homeostatic enzymatic defense enzymes.

#### Enzymatic defense

##### SOD and catalase

Like GSH, superoxide SOD neutralizes superoxide anion O${}_{2}^{•-}$. In eukaryotes there are two species of SOD enzyme, copper-zinc SOD and manganese SOD (SOD_CuZn_ and SOD_Mn_). Conversion of O${}_{2}^{•-}$ to H_2_O_2_ is performed by the cyclic oxidation of the Cu^2+^ or Mn^2+^ transition metal ion localized in the SOD active site (42, 47). Enzymatic antioxidant defense cannot rely solely on SOD, because that would result in markedly increased tissue damage due to high accumulated levels of intracellular H_2_O_2_, the substrate for production of ^•^OH via the Fenton reaction. Terminal transformation of H_2_O_2_ to H_2_O circumvents H_2_O_2_ accumulation, and is performed by the CAT enzyme.

##### Homeostatic regulation of GSH levels

Glutathione peroxidase (GPx), glutathione reductase (GR), glutathione S-transferase (GST), and glucose-6-phosphate dehydrogenase (G6PD) are responsible for recycling of oxidized glutathione (GSSG) back to reduced glutathione (GSH). It is by these means that the GSH molecule neutralize attacks by additional ROS (42, 43).

### Reactive oxygen species-mediated damage to intracellular macromolecules

In this section we provide an overview of the types of cellular macromolecules that are damaged by ROS in mammalian cells including endometriotic cells, sperm, oocytes, and embryos, and other cell types.

#### Point mutations

Genomic DNA, mitochondrial DNA, and cellular RNA species can be attacked by ^•^OH (53, 54). O${}_{2}^{•-}$ and H_2_O_2_ do not attack DNA. ^•^OH reacts with purine and pyrimidine bases in DNA and RNA (43). ^•^OH attacks thymine or deoxyguanosine bases to generate 5-hydroxy-6-hydrothymine or 8-hydroxy-2'-deoxyguanosine (8-OHdG). Measurements of 8-OHdG levels are among the most widely utilized indices of DNA oxidation. Oxidative DNA damage results in mutations that generate dysfunctional protein gene products and altered replication and transcription of crucial genes (44, 53–55).

#### Oxidative stress-induced lipid peroxidation

Lipid peroxidation occurs primarily via peroxidation of unsaturated fatty acids, although saturated fatty acids and membrane cholesterol can also undergo peroxidation. The lipid peroxidation process is primarily initiated by ROS (mostly ^•^OH) (56). The peroxidized lipid radical reaction product is very unstable and covalently reacts with oxygen to create peroxyl radical. This triggers a chain reaction when the peroxyl radical takes hydrogen from another fatty acid, producing a new lipid radical and a lipid peroxide, and on in a sequence, a process termed propagation. The reaction is only terminated once two radicals react with one another or with an antioxidant molecule (43, 44, 57, 58).

#### Damage to proteins

ROS reacts with proteins and disrupts protein folding (44). This results in perturbations in biochemical activities of enzymes, transport proteins, structural proteins, receptors and other proteins such as enzymes that control iron and calcium homeostasis. ^•^OH is the predominant ROS species that causes oxidative damage to proteins. It attacks the α hydrogen atom of an amino acid residue to form a carbon-centered radical that reacts with oxygen to generate an alkyl-peroxyl radical and then in two steps an alkoxyl radical. Amino acid side chains of proteins can be also attacked. Cysteine and methionine residues are specifically susceptible to oxidation.

### ROS damage to oocytes, sperm, and embryos, and prevention by antioxidants

#### Oocytes

Aberrations in microtubule integrity, abundance, and alignment at the metaphase plate have been shown to increase susceptibility to errors in meiotic chromosome segregation in animal oocytes, and may do so in human oocytes, and embryos (59–64). ROS damages oocyte spindle microtubules and also blocks formation of new spindle microtubules (65). Several pathways have been found by which ROS promotes damage and disassembly of meiotic spindle microtubules in oocytes (64):

- Stability of the spindle is dependent on maintaining the dynamic equilibrium between polymerization (assembly) and depolymerization (disassembly) of tubulin subunits to maintain intact spindle microtubules. ROS inactivate protein kinases that phosphorylate microtubule-associated proteins (MAPs) that maintain this dynamic equilibrium (65).
- ROS impedes mitochondrial respiration, reducing the rate of ATP synthesis via destruction of mtDNA (65, 66). This can be expected to result in GTP depletion. GTP is an essential co-factor for microtubule assembly (67). ROS is thereby expected to impede microtubule assembly.
- A high intracellular ratio of reduced glutathione to oxidized glutathione (GSH/GSSG ratio) is protective of microtubules in their polymerized state by the preventing oxidation of cysteine residues in tubulin subunits. Conversely, a low ratio is permissive for tubulin subunit oxidation, and thus favors microtubule disassembly. Fewer intact microtubules are available to faithfully orchestrate the process of meiotic chromosome segregation in the oocyte (65).

Experimental evidence for oxidative stress-induced chromosome segregation errors in *Drosophila melanogaster* oocytes was published by Perkins et al. (68). Knock-down of the SOD genes encoding *SOD1* and *SOD2* induces meiotic chromosome nondisjunction. Zuelke et al. demonstrated that GSH plays an important role in hamster oocyte spindle function. Depletion of GSH with the GSH oxidant diamin disrupted the meiotic spindle apparatus (69). A number of studies have shown chromosome and spindle misalignments and aneuploidy in oocytes of mice exposed to ROS (70–74). For example, germinal vesicle (GV) mouse oocytes were treated with different H_2_O_2_ concentrations during *in vitro* maturation displayed concentration dependent increased incidences of misaligned chromosomes, spindle abnormalities, and aneuploidy (75).

A number of studies have shown the ability of antioxidant treatments to protect mouse oocytes from chromosome and spindle misalignments and aneuploidy. Vitamin C, vitamin E, α-lipoic acid and acetyl-carnitine, resveratrol, and N-acetyl cysteine protect oocytes from genomic DNA damage, mitochondrial dysfunction, abnormal spindle morphology, and chromosome misalignments (73–78).

He et al. reported that mouse oocyte mitochondria produce the antioxidant melatonin (79). Treatment of IVM mouse oocytes with melatonin reduces ROS production and inhibits 8-oxodG formation. It increases mitochondrial mtDNA copy number, decreases the fraction of oocytes with spindle aberrations and blastocysts from fertilized oocytes that were treated with melatonin are of higher quality.

#### Sperm

The coupled pro-oxidant enzyme system xanthine-xanthine oxidase (XXO), generates O${}_{2}^{•-}$ and H_2_O_2_ (80). Human spermatozoa treated vitro with XXO show significantly increased DNA fragmentation by TUNEL analyses. Co-incubation with antioxidants significantly reduced rates of DNA fragmentation.

Dietary antioxidant supplementation of men improves their semen parameters. Vitamins A and E reduce sperm DNA fragmentation, pyncogynol improves sperm quality and increases testosterone levels, and alpha lipoic acid improves sperm motility (81–83). These data point to ROS damage as an important determinant of genomic integrity and viability of the male gamete.

Burruel et al. found that XXO increases lipid peroxidation of Rhesus Macaque sperm and it decreases sperm motility (84, 85). They also found adverse outcomes for the embryos from the sperm exposed to ROS damage. MII embryos from macaque oocytes fertilized with these sperm displayed lower rates of embryo development than those that had been fertilized with control sperm, and the embryos ultimately underwent fragmentation and permanent mitotic arrest. In preliminary observations, abnormal mitotic spindles and DNA fragmentation of embryos was also observed, and Burruel et al. suggest that these abnormalities may play roles in causing arrest of the embryos. In a similar study with bull sperm, Barbato et al. also found that XXO increased sperm DNA fragmentation, and it decreased sperm motility and fertilization rates. Blastocysts grown from oocytes fertilized with sperm that had been exposed to XXO displayed decreased blastocyst developmental competence by increasing blastomere DNA fragmentation (86). Treatment with antioxidant Coenzyme Q10 and cofactors mitigated these effects.

#### Embryos

ROS impedes embryo development and causes embryotoxicity and teratogenesis. Cultured mouse zygotes were treated with H_2_O_2_ in dose response studies (87, 88). H_2_O_2_-treated zygotes had elevated levels of ROS, as well as increased oxidative damage, activation of the G2/M checkpoint, decreased cleavage and blastocyst rates, and increased rates of apoptosis. In other studies, phenytoin was used as a reagent to treat mouse embryos because it causes oxidation of DNA, proteins, and lipids, likely due to increased production of ^•^OH radicals (89, 90). Cultured gestational day 9.5 mouse embryos treated with phenytoin displayed substantially higher levels of 8-OH-2′-dG DNA modifications than vehicle-treated control embryos and significant developmental damage. Media supplemented with SOD and CAT enzymes reversed 8-OH-2′-dG DNA modifications and prevented morphological anomalies. GSH, vitamin E, and iron chelators also reduce macromolecular embryonal damage and teratogenicity (90).

### Part IV: reactive oxygen species and oxidative damage in endometriosis

#### Proximity of gametes and embryos to ROS from inflammatory response

ROS are in female oviductal fluid and PF and function as normal signaling modulators of ovulation, embryo development and implantation (91, 92). Endometriomal implants with proximity to the ovary include those that are directly on the surface of the ovary itself, and those that reside in the peritoneum and in the fallopian tubes. These explants have the potential to expose the growing follicle, the oocyte, sperm, and embryos to high levels of ROS that are generated by inflammation.

Endometriomas create physical blockages and can impede normal fluid flow and motility in the oviduct, the peritoneum and the vicinity of the ovary, facilitating the buildup of refluxed blood and its components. These include apoptotic endometrial tissue and desquamated menstrual cells. They trigger an inflammatory response that activates macrophages and neutrophils (93). Elevated serum IL-6 and TNF-α levels indicative of increased inflammatory activity have been observed in patients with endometriosis. Singh et al. found elevation of numerous cytokines within follicles of endometriosis patients compared to those of normal ovulating women, with intrafollicular IL-8, IL-12, and adrenomedullin inversely correlated with rates of oocyte maturation and embryo grade (94). Elevated pro-inflammatory cytokines IL-1β, TNF-α, and IL-6 are indicative of an inflammatory microenvironment for the follicles and gametes and embryos of endometriosis patients (95–98). IL-6 and TNF-α, activate intracellular signal molecules to induce production of O${}_{2}^{•-}$ and H_2_O_2_ during the pathogenesis of endometriosis (99) via the respiratory burst NADPH oxidase system (39). Superoxide anion is converted to peroxide by local SOD enzymes (29, 100). Levels of O${}_{2}^{•-}$ and H_2_O_2_ produced by these inflammatory responses in the peritoneal, ovarian and oviductal environments of endometriomal implants are significantly higher than those which are generally encountered in their healthy counterparts (40, 93). Elevated ROS levels are also observed in granulosa cells from endometriosis patients (101). Pathological increases in local concentrations of ROS cause oxidative stress (102).

#### Pernicious effects of refluxed blood and the balance of pro-oxidant and antioxidant forces: the fenton reaction

In addition to inflammatory components, accumulated refluxed menstrual blood contains numerous lysed erythrocytes. In serum of women with endometriosis, the concentration of iron is significantly increased by 1.98-fold (103), and it is highly likely that the iron concentrations at local implant sites are elevated even further. This along with the elevated level of peroxide is a “perfect storm” for generation of hydroxyl radical, the most reactive and destructive form of ROS. Peroxide is converted to hydroxyl radical ^•^OH, which is catalyzed by the excess localized iron via the Fenton reaction. It is by these means that the interplay of these free radical sources is thought to cause ROS damage by ^•^OH to the surrounding tissues including the peritoneum, oviduct, ovary, and the follicle, oocyte, sperm and embryos (29, 84, 85, 103–113).

#### Other types of oxidative damage in endometriosis

Lipid peroxidation is a sensitive index of oxidative stress induced by ROS. Levels of the peroxidized lipid malondialdehyde (MDA) are significantly elevated in FF of patients suffering from endometriosis compared to control patients with tubal or minimal male infertility (29, 103, 105, 106, 111). Glutathione-S-transferase (GST) enzymes exert antioxidant effects by coupling ROS to glutathione (114). GST concentrations decline significantly in serum and PF as a function of increased severity of endometriosis (115). Indeed, a decrease in total antioxidant capacity is observed in sera of patients with endometriosis (111, 113).

Heat shock proteins (HSPs) play a role of central importance in protecting cells from damage by inflammation, oxidative stress, and other causes (116). Dysregulation of HSPs is implicated in endometriosis. Heat shock chaperone proteins such as Hsp70 carry out key functions in the repair of cellular injury including reversal of protein misfolding that occur due to oxidative damage. Elevated serum HSP70 levels are found in women with endometriosis compared to control women (117). Apoptosis is triggered when ROS-mediated cellular injury is sufficiently severe that it cannot be repaired.

#### Balance of pro-oxidant and anti-oxidants in endometriosis

Epithelial cells from deep endometriotic lesions proliferate more rapidly than their normal endometrial counterparts. They too are sources of ROS (112). This includes increased proliferation of endometriotic stromal cells. These proliferating cells produce significantly more O${}_{2}^{•-}$ and H_2_O_2_ than their normal epithelial and stromal counterparts (108, 112). Treatment with the potent anti-oxidant N-acetyl cysteine (NAC), a precursor of reduced glutathione (GSH), significantly inhibits proliferation of human endometriotic cells. This may implicate ROS as an autocrine growth factor that promotes the pathogenesis of endometriosis.

Endometriotic stromal and epithelial samples display a significant increase in levels of SOD and GSH. This would be expected to produce an excess of peroxide (112). Singh et al. found 1.9-fold lower specific activity of CAT enzyme in FF of women with endometriosis (29). Singh et al. also found significantly reduced specific activity of CAT (1.9-fold lower), glutathione peroxidase (GPx; 2.7-fold lower), and glutathione reductase (GR; 5-fold lower) in endometriosis tissue samples from patients with endometriosis. Given that CAT, GPx, and GR catalyze the terminal reduction of peroxide to water to prevent accumulation of hydroxyl radicals, this is a potentially ominous finding. Taken together these results suggest another perfect storm, whereby levels of ^•^OH are markedly increased in FF, risking increased damage to the follicle and the oocyte, although as discussed above, intracellular hydroxyl radical concentration is not directly measurable due to its extreme chemical reactivity.

### Oxidative stress caused by the microenvironment in endometriosis and its effects on the quality of oocytes, sperm, and embryos

As discussed above, the data from the literature demonstrate lower implantation rates, lower pregnancy rates, and higher miscarriages rates in IVF patients with endometriosis. These patients exhibit lower numbers of retrieved oocytes, lower quality of oocytes, lower number of embryos and quality of embryos, and lower rates of blastocyst formation (24–30). Key aspects of the pathogenesis of endometriosis including diminished fertility have been recapitulated in surgically-induced rodent models of endometriosis (118–120). The mice ovulates the same number of MII oocytes, but a lower percentage is morphologically normal, and the yield of zygotes per dam is diminished. Rats with surgically induced endometriosis have reduced fecundity, develop fewer ovarian follicles, and have morphologically abnormal MII oocytes with increased rates of meiotic chromosome and spindle misalignments compared to sham-operated rats. Day 3 embryos have higher rates of fragmentation and cleavage delayed or arrested cleavage (121). One would predict that semen parameters would also be adversely affected in these models, although Pubmed searches indicate that this study has yet to be performed.

A key hypothesis we are studying here is that in endometriosis, a pro-oxidant microenvironment in the local vicinity of the follicle created by an unbalanced redox state detrimentally impacts the quality of oocytes, sperm, and embryos, hampering their cellular function, jeopardizing their viability, and that this is a fundamental underlying cause of diminished fertility with endometriosis. Since FF and PF comprise key constituents of the local environment to which gametes and preimplantation embryos are exposed, a number of studies have investigated ROS activities and oxidative stress response to FF and PF from IVF patients with endometriosis, and their abilities to damage gametes and embryos.

Mass spectroscopy proteomics of FF from women with endometriosis indicates elevated oxidative stress status (122). Pro-inflammatory cytokines in FF and PF from women with endometriosis are elevated compared to PF and FF of control women (40, 123–126). FF from women with endometriosis has significantly higher levels than FF from control women of advanced oxidation protein products, novel markers of oxidative stress (127). 8-OHdG modifications in DNA are elevated in FF and PF from women with Stage I/II and Stage III/IV endometriosis compared to control patients (54, 105, 128). 8-OHdG elevation may be due to both increased load of ROS and decreased expression of OGG1, the base excision repair gene that excises and repairs single base mutations, which in fact was found in endometriosis patients (105). Studies by Da Broi et al. and by Huang et al. found less antioxidant activity in FF of patients with endometriosis than in control patients with tubal infertility (129, 130). Some controversy remains, however, since a single study by Nakagawa et al. found no elevation of total oxidative stress indices in FF between patients with a unilateral endometrioma and healthy patients (131).

Bovine and mouse IVM MII oocytes were incubated in PF and FF from women with endometriosis vs. from control women to compare their effects on oocyte quality. Oocytes were from animals due to ethical prohibitions against studies that could purposefully damage human oocytes. PF and FF from women with endometriosis caused significantly greater chromosome and spindle misalignments in bovine and mouse MII oocytes than PF and FF from control women (109, 110, 128, 132, 133).

Sperm from healthy men was co-incubated with PF from patients with endometriosis vs. healthy control patients. TUNEL analyses showed significantly more DNA fragmentation of the DNA from sperm incubated with PF from endometriosis patients than from control patients (109). There was no effect on morphology by conventional criteria. In other studies, sperm motility was not impacted by PF from endometriosis patients (134). Fertilization rates of mouse oocytes by mouse sperm co-incubated in PF from endometriosis patients declined significantly from their fertilization rates in PF from control patients (135). Mouse embryos cultured in PF or FF from endometriosis patients show decreased cell number and rates of growth, decreased cleavage and blastulation rates, and increased rates of DNA fragmentation, developmental arrest, embryo toxicity, and apoptosis than mouse embryos cultured in PF or FF from control patients (109, 110, 135–141).

Peritoneal and follicular fluids are complex mixtures with thousands of diverse components. If elevated ROS and/or lower antioxidant activities are responsible for the damage done to gametes and embryos incubated in PF and FF of endometriosis patients, then the addition of antioxidants to PF and FF incubation medium should decrease rates of gamete and embryo damage. Supplementation of PF and FF media from women with endometriosis with N-acetyl cysteine (NAC) or L-carnitine (LC) antioxidants reduced rates of chromosome and spindle abnormalities in mouse and bovine MII oocytes (110, 132). LC also prevented apoptosis of mouse embryos incubated in PF of endometriosis patients (110, 142). The data with NAC indicate that antioxidants can improve egg quality and they support the hypothesis that pro-oxidant activity in FF comprise key components responsible for its detrimental effects on egg quality. As L-carnitine has diverse biological activities, additional studies will be required to determine whether its beneficial effects are attributable to its antioxidant properties. Future studies investigating impacts on oocyte and embryo quality by additional anti-oxidant supplements will be illuminating, as will experiments to determine whether antioxidants can prevent detrimental effects of PF and FF on sperm motility and morphology.

### Part V: could the altered microenvironment in endometriosis lead to oocyte and fetal aneuploidy?

Aberrations in oocyte spindle integrity and chromosome misalignments at the metaphase plate are predictive of impending aneuploidy (143). Given that PF and FF from women with endometriosis cause chromosome and spindle misalignments in bovine and mouse oocytes, does this mean that endometriosis causes oocyte and fetal aneuploidy?

There are several limitations to the conclusions that can be drawn from the studies with PF and FF. Some studies employed relatively small sample sizes. Experiments analyzed impacts of endometriotic human fluids on healthy oocytes from heterologous animal species. The experimental design is ethical by not exposing human oocytes to treatment that is expected to have an adverse effect. However, it is possible that human oocytes will not be sensitive to the same treatments as animal oocytes. Finally, whereas chromosome and spindle misalignments are likely correlated with impending meiotic aneuploidy, the correlation with aneuploidy is not universal (144).

Rajani et al. found that human IVF-ET patients with endometriosis (*n* = 56) had comparable numbers of retrieved oocytes, mature MII oocytes, and rates of visualized spindles, vs. invisible spindles, to women with tubal infertility (*n* = 63) (145). This is significant since the visualization of a spindle is predictive of embryonic developmental competence (146). However, suitable methodology for visualization of spindles was not demonstrated since no pictures of oocyte spindles were presented. In addition, spindle misalignments were not scored, so the question of aneuploidy was not addressed. Fertilization rates, yields per patient of fertilized embryos, and embryo grades were also comparable between the test groups. While there was no significant difference between pregnancy rates, there was a trend toward lower pregnancy rates in patients with endometriosis. These data suggest no significant differences in oocyte or embryo quantity or quality or pregnancy achievement between the test groups. However, we suggest that patients with male infertility would have been a better control group than patients with tubal infertility, given a theoretical potential for pathogenic processes in the ovarian microenvironment for the latter group. In addition, the numbers of patients per test group were small enough that even robust differences may have been missed, according to a chi square power analysis of proportions performed by the authors of this review.

Gianaroli et al. performed PGS analyses of polar bodies biopsied from oocytes from patients undergoing IVF treatment for diverse infertility conditions, including endometriosis (147). Oocytes were retrieved from patients from diverse age groups with different infertility conditions and stimulation protocols. FISH analyses were performed six for chromosomes for which trisomies are most commonly observed –chromosomes 13, 15, 16, 18, 21, and 22. A total of 3,816 oocytes from 544 patients were analyzed. Multivariate regression analyses were performed to evaluate which input variables (i.e., type of infertility, treatment protocol, age group) are most strongly associated with chromosomal abnormalities compared with a control group. Regression coefficients were calculated for the proportion of normal oocytes for each group divided by the number of diagnosed oocytes. A significant relationship was observed between the incidence of endometriosis and the occurrence of errors in any of the six chromosomes (Regression coefficient = −12.223; *P* < 0.01).

Direct comparison of the fraction of oocytes with one or more aneuploid chromosomes was performed for PB1 from patients in this study with endometriosis (38 cycles) vs. control subjects (58 cycles). The control group was primarily comprised of patients with male factor or idiopathic infertility. 210 of 228 biopsied oocytes were diagnosed in the endometriosis group and 382 of 426 oocytes were diagnosed in the control group. A significantly higher fraction of polar bodies in the endometriosis group exhibited aneuploidy compared to the control group, with 112/210 polar bodies (53%) with a one or more aneuploid chromosomes in the endometriosis group vs. 159/382 polar bodies (42%) in the control group (*P* = 0.0075, two sided Fisher exact test; L. Gianaroli, personal communication). In control studies with 100 donated oocytes, there was 95% concordance between FISH results between PB1 polar bodies and the oocytes from which they were derived. There was no significant difference in age between the test groups (34.5 years for the endometriosis group and 34.4 years for the control group, *P* > 0.05). These data indicate with 67% power a higher rate of aneuploidy in oocytes from patients with endometriosis than control patients (chi square test of two proportions). Four-hundred oocytes per test group would be needed to achieve 80% power. Comprehensive chromosomal screening (CCS) of all 24 human chromosomes might have discerned significant differences with the number of oocytes that were available for biopsy.

A very large IVF-ET study by Juneau et al. (148) compared preimplantation embryos by preimplantation genetic testing for aneuploidy (PGT-A). It compared patients with endometriosis (*n* = 305 patients, 1,880 blastocysts analyzed) to an aggregate population of patients without endometriosis who suffered all other forms of infertility (*n* = 3,798 patients, 23,054 blastocysts analyzed). Endometriosis patients had fewer oocytes, fewer 2PN embryos, and a nearly significant decline in usable blastocysts (regarded suitable for transfer). Blastocyst PGT-A for all 24 human chromosomes revealed no significant difference in the rates of aneuploidy between all tranched age groups for endometriosis vs. control patients (*P* > 0.05).

The results of the Juneau et al. study are validated by its large sample size, with the substantial ability to detect differences in aneuploidy rates between test groups, with well over >80% statistical power (Chi square test of two proportions). An actual incidence of oocyte aneuploidy that is higher in the endometriosis patients cannot be ruled out, because attrition of a fraction of aneuploid embryos at earlier growth stages would not have been detected by PGT-A of blastocysts. The control group was a heterogeneous patient population with a variety of infertility disease processes including uterine factor, anovulatory infertility, and tubal factor, each of which could have their own pathogenic process, some perhaps including aneuploidy. PGT-A of blastocysts from women with primarily male factor infertility may have provided a better control. For both Gianaroli et al. and Juneau et al., the severity of endometriosis was not clarified, and without laparoscopic confirmation, the control groups may have included a number of patients with endometriosis.

Overall, the data in the literature demonstrate that endometriosis causes a decline in the quality of oocytes and embryos. Gianaroli et al. provide evidence that endometriosis may cause aneuploidy in the human oocyte, although larger sample sizes are needed to substantiate this. The best data that are available demonstrate that endometriosis is not associated with aneuploidy of embryos that reach the blastocyst stage.

### Part VI: summary and concluding remarks

**Figure 1:**
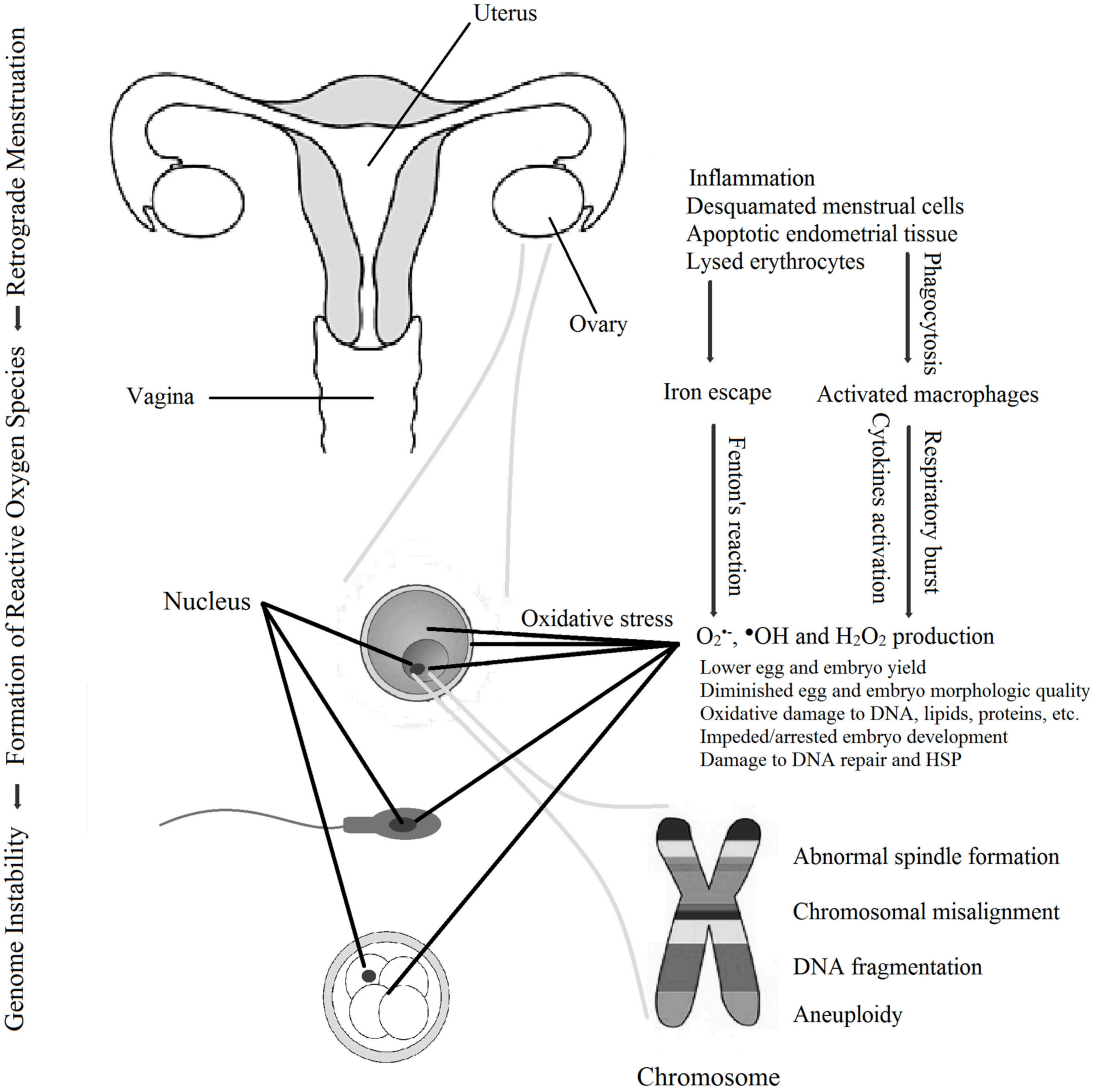
Schematic working model for the means by which reactive oxygen species in endometriosis promote decline in the quality of oocytes, sperm and embryos to cause infertility. Reactive oxygen species-induced damage also includes damage to membranes, mitochondria, and other organelles (not shown).

This last section summarizes a working model characterizing modes of action by which ROS produced in endometriosis has adverse impact on the quality of oocytes, sperm and embryos to impede fertility (Figure 1). Endometriotic tissues implanted in the peritoneal space, fallopian tube, and/or on the ovary have proximity to follicles as they grow, and to oocytes, sperm and embryos as they transit through the fallopian tube. These endometriotic tissues are also in direct physical contact with refluxed blood and tissue debris that arrive by the process of retrograde menstruation. This combination triggers potent inflammatory stimuli *via* macrophage and neutrophil activation and accompanying release of pro-inflammatory cytokines (93, 95–97). This elicits copious production of ROS superoxide anion and peroxide by the inflammatory cells (102, 149). Under these uncontrolled conditions, the concentrations of ROS exceed the capacity of local antioxidant molecules neutralize them (29, 112, 115, 150), resulting in a buildup of ROS O${}_{2}^{•-}$ and H_2_O_2_, and also malondialdehyde, at the site of the ovary and in follicular fluid and peritoneal fluid (29, 103, 105, 106, 108, 111, 112). Transitional iron Fe^3+^ released from the stagnated refluxed erythrocytes magnifies the damage by driving production of hydroxyl radical ^•^OH^.^ from peroxide via the Fenton reaction (103). O${}_{2}^{•-}$ and ^•^OH confer damage to DNA, RNA, carbohydrates, proteins, and lipids that comprise cellular organelles in oocytes, sperm, and embryos (43, 48, 109, 110).

Among the most pernicious effects of ROS in women with endometriosis is the extent to which damage to gametes and embryos can be self-perpetuating and even irreversible, by damaging components of the cell that are responsible for information coding and repair of cellular damage. ^•^OH. as the most potently reactive ROS species damages DNA by causing ^•^OH. causes point mutations, and DNA breaks that cause DNA fragmentation. These damages disrupt the integrity of the genetic code and inheritance for the mitochondrial and nuclear genomes. ^•^OH as well O${}_{2}^{•-}$ and H_2_O_2_, attack intracellular proteins that maintain the fidelity of DNA replication, and the ability of cells to repair intracellular protein damage. Although there is a robust heat shock response of Hsp70 in endometriosis (117), ROS damages heat shock proteins impeding HSP-mediated re-folding of polypeptides misfolded and otherwise damaged due to oxidative stress. This disruption of DNA replication and repair and HSP protein refolding can trigger mitotic arrest and even apoptosis when injury is severe.

Patients with endometriosis have reduced yields of oocytes. A pathway by which this occurs is suggested in a recent study by Hamdan et al., who found that ROS from human FF of patients with endometriosis damaged mouse oocyte DNA and triggered arrest of meiosis in MI via the DNA damage checkpoint (151). MI resumed upon addition of ROS scavengers to the FF oocyte incubation medium. Overall, irreparable damage to membranous organelles, spindle microtubules, cellular DNA, and the heat shock system itself leads to profound dysfunction of gametes and embryos and even apoptotic cell death, all of which adversely impact fertility (84, 85, 107, 109, 110, 152, 153).

A central role for ROS in the decline of gamete and embryo quality in patients with endometriosis is supported by a series of observations. (i) Eggs, sperm and embryos exhibit poorer quality after exposure *in vitro* to exogenous ROS, both by morphological and functional criteria. (ii) Gametes and embryos from patients with endometriosis are exposed to an ROS-rich pro-oxidant microenvironment in the fallopian tubes, the ovary, and the follicle. (iii) The quality of sperm, oocytes, and embryos declines significantly upon exposure to PF and FF from patients with endometriosis. (iv) Incubation of gametes and embryos in PF or FF supplemented with antioxidants prevents much of this damage. These data demonstrate that ROS generated in endometriosis is largely responsible for the adverse effect of the endometriotic microenvironment on gamete and embryo quality.

As cited above, a key manifestation of oxidative damage to oocytes in endometriosis is comprised by aberrations in the organization of oocyte meiotic spindles and chromosomes, a key morphologic marker for ROS damage and a widely regarded harbinger of impending oocyte and fetal aneuploidy (62, 84, 85). However while spindle misalignments have been widely reported in endometriosis studies involving mouse and bovine oocytes, spindles in human oocytes from patients with endometriosis have yet to be visualized with sufficient detail to determine whether they have misalignments (154). Paradoxically, work with polar bodies from endometriosis patients indicates increased rates of aneuploidy in patients with endometriosis, with statistically larger sample sizes needed to authenticate this conclusion, while definitive data demonstrate that embryos from endometriosis patients do not (147, 148). Thus, the extent to which endometriosis causes oocyte aneuploidy remains to be answered definitively, and if it does, the extent to which the aneuploidy is caused by ROS in endometriosis will need to be addressed.

#### Treatment horizons

Given the *in vitro* studies showing that antioxidant supplementation of peritoneal and follicular fluids mitigates damage to human sperm, oocytes and embryos, it is logical to predict that *in vivo* treatments with antioxidants will improve parameters of fertility in endometriosis. A number of antioxidants have been tested. Vitamins C and E (155, 156), N-acetyl cysteine (NAC) (112, 157), resveratrol (58–162), and melatonin (163–166) have variously been shown to reduce the numbers and sizes of endometrotic implants in animal studies. A single study with insufficient statistical power was performed testing the effects of vitamins C and E in humans, and no clinical studies of resveratrol or melatonin effects on endometriotic lesions in women were found in Pubmed searches as of July 2108. However, in a studying employing the human-like SCID mouse model, melatonin induced regression of transplanted human endometriotic lesions (166).

To date the studies with NAC have progressed the most toward therapeutic potential. A combination of NAC with α-lipoic acid (also an antioxidant) and bromelain prevents upregulation of the VCAM1 inflammatory marker, promotes apoptosis of endometriotic but not normal uterine cells, and reduces the size and number of endometriotic lesions in mice (167). A promising clinical trial of women with endometriosis showed decreased diameter and volume, decreased number, and even disappearance of endometriotic lesions after treatment for 3 months with NAC (168). These studies demonstrate a key role for ROS in the growth and/or maintenance of endometriotic lesions. ROS are also essential mediators of angiogenesis, degeneration of extracellular matrix, anti-apoptotic processes, and cellular adhesion processes that participate in the formation and maintenance of endometriotic implants (169). In this way, ROS have at least an indirect impact on the quality of gametes and embryos. As discussed earlier, NAC and other antioxidants prevent chromosome and spindle misalignments and aneuploidy in mouse oocytes and embryos. Future studies will further elucidate the roles of ROS in gametic and embryonic dysfunction and aneuploidy in infertility caused by endometriosis, and therapeutic regimens dedicated to mitigating the impact of ROS on these processes will continue to be developed.

## Author contributions

GM, LB and AT contributed to develop the theory, to the design and implementation of the research and to the writing of the manuscript.

### Conflict of interest statement

LB is employed by company Pregmama, LLC, Gaithersburg, MD, United States. The remaining authors declare that the research was conducted in the absence of any commercial or financial relationships that could be construed as a potential conflict of interest.

## Abbreviations

8-OHdG: 8-hydroxy-2'-deoxyguanosine
AMH: anti-Müllerian hormone
ART: assisted reproductive technology
ASRM: American Society of Reproductive Medicine
ATP: adenosine triphosphate
CAT: catalase
CCS: comprehensive chromosomal screening
FISH: fluorescence *in situ* hybridization
FF: follicular fluid
G6PD: glucose-6-phosphate dehydrogenase
GPx: glutathione peroxidase
GR: glutathione reductase
GSH: glutathione
GST: glutathione S-transferase
H_2_O_2_: hydrogen peroxide
ICSI: intracytoplasmic sperm injection
IL-6: interleukin 6
IVF: *in vitro* fertilization
LC: L-carnitine
MDA: malondialdehyde
mtDNA: mitochondrial DNA
NAC: N-acetyl cysteine
O${}_{2}^{•-}$: superoxide anion radical
^•^OH: hydroxyl radical
PF: peritoneal fluid
PGS: preimplantation genetic selection
PGT-A: preimplantation genetic testing for aneuploidy
PN: pronucleus
ROS: reactive oxygen species
SOD: superoxide dismutase
TNF: tumor necrosis factor
XXO: xanthine oxidase.
